# Review of Oncology (Oxford Core Text), 2^nd ^Edition, by Max Watson, Ann Barrett, Roy Spence, and Chris Twelves

**DOI:** 10.1186/1477-7800-5-5

**Published:** 2008-02-29

**Authors:** Chung T Lim, Chung S Lim

**Affiliations:** 1 St. Bartholomew and the Royal London School of Medicine and Dentistry, Queen Mary College, University of London, UK; 2 Department of Vascular Surgery, Charing Cross Hospital, Fulham Palace Road, London W6 8RF, UK

## Review

This is the 'Oncology' theme book from the popular collection of core textbooks of various medical specialties published by the Oxford University Press (the others include 'Endocrinology', 'Neurology' and 'Haematology'). The first edition was published in 2001, with the second edition five years later. Compared to the first, this edition contains several updated facts related to the ever-changing field of oncology. It is written by four experts who have many other publications related to oncology.

**Figure 1:**
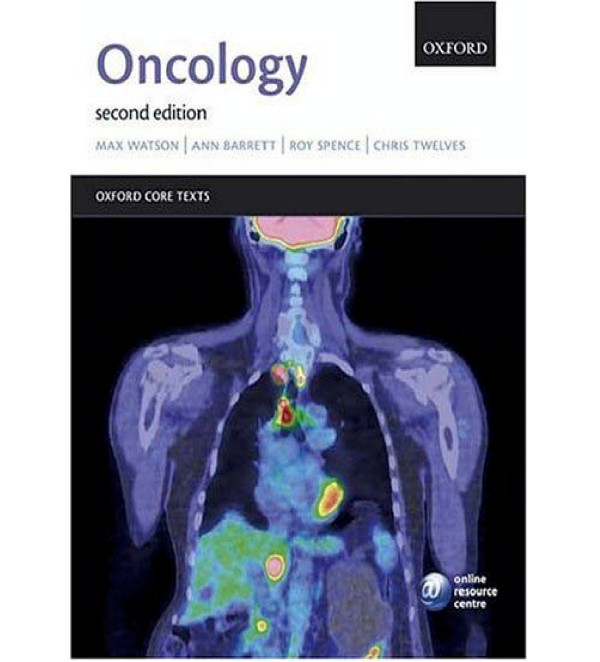
Front Cover of Oncology (Oxford Core Text), 2^nd ^Edition, by Max Watson, Ann Barrett, Roy Spence, and Chris Twelves.

This book definitely stands out among the medical students and junior doctors. It measures 246 mm × 189 mm, with 212 pages (Figure 1). Therefore it is fairly easy to carry around especially for a textbook. The layout of the book is simple, with two-colour presentation (blue and black) accompanied by pictures and diagrams to aid study. The topics are arranged accordingly with a detailed index at the end of the book which allows easy referencing. There are 19 chapters, with the first seven explaining the general features of oncology, including the epidemiology, pathogenesis, diagnosis, staging, and treatment of cancers. The authors then dedicated three separate chapters to the three major cancers in the Western world, i.e. the lung, colorectal, and breast cancers. The remaining chapters discuss the other cancers which are classified based on the bodily systems such as the genitourinary system, central nervous system, and head and neck.

The language used is simple and easy to understand. The facts are well presented in sentences and paragraphs that flow and hence enjoyable reading. Many important facts are also presented in point forms, making learning more efficient. The summary at the end of each chapter enables the reader to reflect on the main points discussed in that chapter. Topping up to these are the 'key facts' and 'stop and think' boxes which encourage the reader to actively participate in the discussion rather than just reading passively.

We would recommend this book particularly to the medical students and junior doctors who are in oncology-related attachments. It provides them a good basic understanding of oncology and yet it does not take too long to read. It is also suitable to the nursing staff and other healthcare professionals who are keen to learn more about oncology to help them in their daily clinical work.

## Competing interests

The author(s) declare that they have no competing interests.

